# Concurrent Acute Limb Ischemia and Pulmonary Embolism in an Adult With Repaired Tetralogy of Fallot: A Case Report

**DOI:** 10.7759/cureus.81665

**Published:** 2025-04-03

**Authors:** Anas A Abu-Wardeh, Abdel G Anabtawi

**Affiliations:** 1 Cardiology, Jordan University of Science and Technology, Irbid, JOR; 2 Interventional Cardiology, First Coast Cardiovascular Institute, Jacksonville, USA

**Keywords:** acute and chronic limb ischemia, acute lower limb ischemia, arterial thromboembolism, interventional cardiology, pulmonary embolism, tetralogy of fallot

## Abstract

Simultaneous acute limb ischemia (ALI) and pulmonary embolism (PE) are extremely rare, with the current literature providing a limited understanding of shared mechanisms. We report a rare case of concurrent ALI and PE presentation in a patient with a history of repaired tetralogy of Fallot (TOF), emphasizing the possible association between congenital heart disease and thromboembolic risk, potentially through residual hemodynamic abnormalities or arrhythmias. The case involves a 48-year-old male with a history of TOF repair in childhood who developed simultaneous ALI and PE approximately 48 years after the surgical repair. The patient presented with sudden right lower extremity pain and swelling; was classified as Rutherford IIa ALI; and underwent urgent endovascular revascularization that included an overnight tissue plasminogen activator infusion and a relook angiogram the next day. During admission, the common embolic etiologies of his presentation were excluded by multiple investigations, including transthoracic echocardiography, thrombophilia panel, and occult malignancy screens. Although the patient had multiple cardiovascular risk factors, including obesity, smoking, and hypertension, it is most likely the history of TOF repair that contributed the most to the concurrent thromboembolic presentation, especially given that TOF repair has been reported to be associated with chronic complications in adult patients, such as persistent right ventricular dysfunction and arrhythmias. A multidisciplinary team of interventional cardiologists, pulmonologists, and hematologists managed the case, reflecting the difficulty of managing concomitant thromboembolic events and monitoring and evaluating thromboembolic risk factors for treated congenital heart disease, most specifically TOF, in adult patients.

## Introduction

Tetralogy of Fallot (TOF) is a type of congenital heart disease (CHD) that presents at birth with four key defects commonly occurring together. Classically, the four defects are ventricular septal defect, right ventricular outflow tract obstruction, aortic override, and right ventricular hypertrophy. TOF occurs in approximately 3 in 10,000 live births and accounts for 7-10% of all CHDs. Treatment is surgical repair with either a complete neonatal repair or staged palliation, followed by subsequent repair in the first six months of life [1].

Venous thromboembolism (VTE), in the form of pulmonary embolism (PE) and deep vein thrombosis (DVT), is characterized by the development of venous clots, a product of Virchow’s triad of stasis, endothelial damage, and hypercoagulability [2,3]. Risk stratification for PE is crucial, and the Pulmonary Embolism Severity Index (PESI) remains the most extensively validated tool for identifying patients at low risk for 30-day mortality [4].

VTE takes on a different context in CHD, where abnormal flow patterns, such as those seen with arrhythmias, can predispose patients to thromboembolic risk [5,6]. While VTE is primarily associated with venous pathology, the simultaneous occurrence of arterial and venous thromboembolism is rare, suggesting a shared pathophysiologic mechanism. This simultaneous occurrence is particularly relevant in CHD, where systemic hypercoagulability (via obesity, smoking, and hypertension), paradoxical embolism (via residual intracardiac shunts), and hemodynamic stasis (due to right ventricular failure or immobilization) contribute to an increased risk of both arterial and venous events [7].

A critical arterial event that may manifest from a shared pathophysiologic mechanism is acute limb ischemia (ALI), classically presenting with the “five Ps”: acute pain, pallor, pulselessness, paresthesia, and paralysis. ALI, which arises when there is an abrupt decrease in a limb’s blood perfusion, can ultimately endanger limb viability. Thus, the clinical presentation of these signs and symptoms should prompt a rapid diagnosis and intervention [8]. There are several categorization schemes for ALI stratification. Bollinger and Graziani take anatomical factors into account, whereas Fontaine and Rutherford focus on symptomatology [9]. Because of its widespread clinical adoption and suitability for acute presentations, Rutherford’s classification, which expands upon Fontaine’s by including objective clinical data, was applied in this instance.

Thromboembolism in repaired TOF patients can manifest as both arterial and venous events, with their predisposition through several related mechanisms. Residual chamber defects, such as persistent right ventricular dilation and dysfunction, alter hemodynamics and increase blood stasis, which may contribute to thrombogenesis [10]. Additionally, arrhythmias are a common late complication of TOF repair, with atrial fibrillation and other arrhythmias significantly increasing in incidence beyond 45 years of age, which may promote thrombus formation due to abnormal atrial contraction [11]. Collectively, these factors create a prothrombotic state that may be responsible for the rare concurrent presentation of arterial and venous thromboembolism in adult survivors of repaired TOF.

This case emphasizes the educational value of recognizing rare yet critical presentations of thromboembolism in adult survivors of congenital heart disease, especially TOF. It highlights the implications for post-TOF surveillance, emphasizing that even decades after surgical repair, patients may develop complex thromboembolic events that challenge standard diagnostic and management protocols. Understanding these mechanisms can enhance early detection and inform tailored therapeutic strategies, ultimately improving patient outcomes.

## Case presentation

This is the case of a 48-year-old male patient with a history of surgically corrected TOF at 11 months of age. However, because the patient had no accessible medical records and no sufficient knowledge of his TOF repair history, information about the type of repair (transannular vs. non-transannular) was not available. His past medical history also included hypertension, hyperlipidemia, obesity (body mass index 41.0 kg/m²), smoking, gastroesophageal reflux disease (GERD), depression, obstructive sleep apnea (OSA), and alcohol abuse. The patient presented with sudden-onset right lower extremity pain and swelling below the knee, lasting four hours. The pain was constant and associated with numbness, tingling, and weakness. It awakened him from sleep and persisted. The patient reported that his leg “gave out” when attempting to walk. He denied any history of previous similar events, DVT, trauma, immobilization, chest pain, syncope, palpitations, hemoptysis, fever, or dyspnea (neither at rest nor exertion). He also denied any history of diabetes mellitus or recent surgeries. His home medications only include paroxetine, omeprazole, and hydrochlorothiazide for managing depression, GERD, and hypertension, respectively. Family history was significant for DVT in his mother, who was on apixaban.

He was transferred via air to a higher-level care facility after receiving heparin and supplemental oxygen (SpO_2_ 90% on presentation) at the referring hospital. He arrived on a nasal cannula (3 L/minute O_2_) but was not on a heparin drip. Chest, abdomen, pelvis, and run-off computed tomography angiogram (CTA) at the referring facility revealed lobar and segmental pulmonary emboli in the left upper and lower lung lobes; embolic arterial disease with occlusive thrombus in the right external iliac arteries; non-occlusive thrombus in the right common and internal iliac arteries; a large splenic infarct; a left renal infarct in the upper pole; and cardiomegaly.

Upon arrival, the patient was alert, oriented, well-built, and in no distress. Vital signs were as follows: temperature, 36.7°C; respiratory rate, 16 breaths/minute; pulse, 76 beats/minute; blood pressure, 131/64 mmHg; and SpO_2_, 93%. Physical examination revealed a cold, non-tender right lower leg with absent pedal pulses. Lungs were clear to auscultation. The patient’s history and hemodynamic stability initially yielded a PESI score of a very low risk mortality stratum [4].

Essential laboratory work, including complete blood count (CBC), comprehensive metabolic panel (CMP), prothrombin time/international normalized ratio (PT/INR), brain natriuretic peptide (BNP), troponins, and arterial blood gas (ABG), revealed normal results except for elevated troponin I (286 pg/mL) and an abnormal ABG suggestive of acute hypoxemic respiratory failure with respiratory alkalosis (pH: 7.49, partial pressure of oxygen (PaO_2_): 53 mmHg, partial pressure of carbon dioxide (PaCO_2_): 34 mmHg). The elevated troponins raised the potential risk of this case and could be suggestive of right heart strain due to acute PE, although BNP was within normal ranges. Acute hypoxemic respiratory failure with respiratory alkalosis was also suggestive of a higher risk of PE.

Electrocardiogram showed normal sinus rhythm with complete right bundle branch block. Venous ultrasonography identified non-occlusive DVT in the left popliteal and gastrocnemius veins. This finding highlighted a concurrent venous and arterial thrombotic burden, distinct from isolated VTE pathways. No DVT was detected in the right lower extremity.

**Figure 1:**
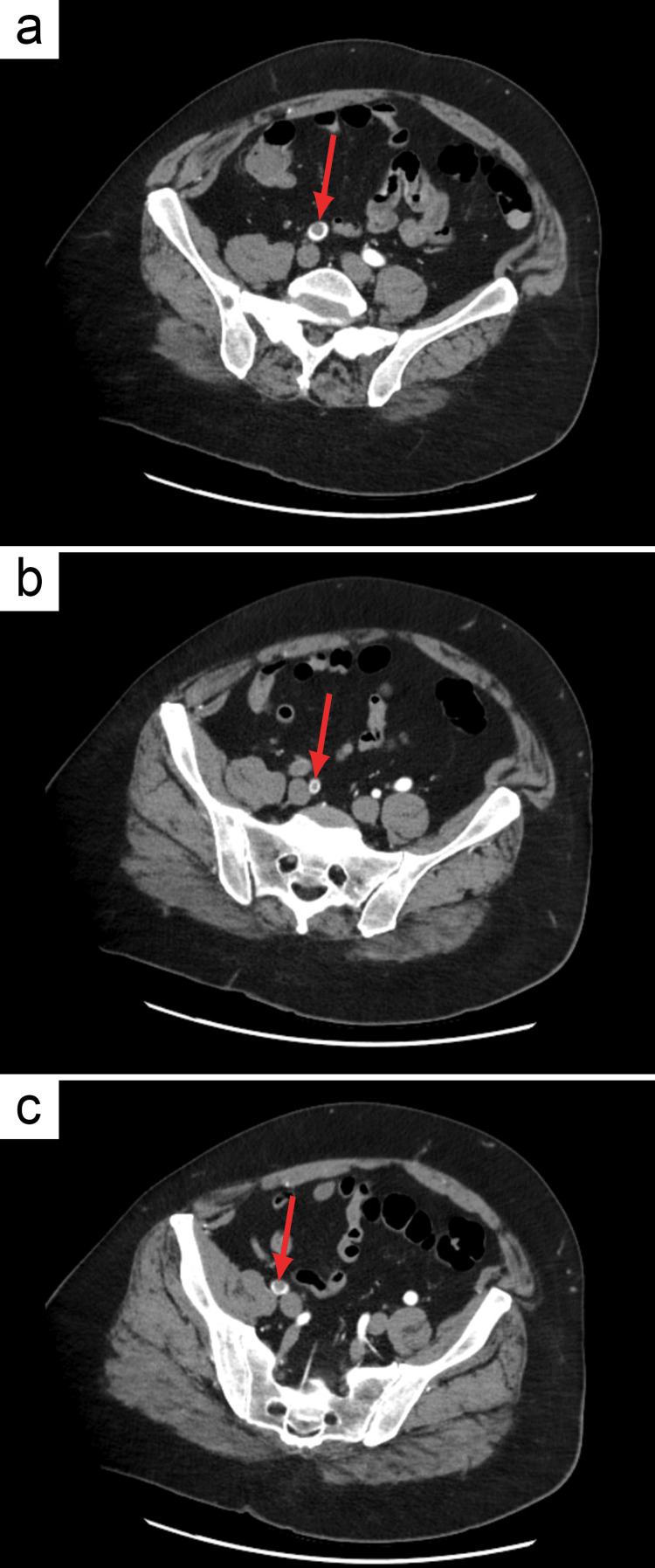
Axial computed tomography angiography of the patient’s abdomen/pelvis. The repeat chest, abdomen, pelvis, and run-off computed tomography angiography confirming acute limb ischemia findings, including thrombus (red arrows) within the iliac vessels. (a) Non-occlusive thrombus in the right common iliac artery. (b) Non-occlusive thrombus in the right internal iliac artery. (c) Occlusive thrombus in the proximal right external iliac artery.

Repeat chest, abdomen, pelvis, and run-off CTA (Figure 1) confirmed ALI findings, including acute non-occlusive thrombus in the right common iliac artery, acute short-segment occlusion of the proximal right external iliac artery with reconstitution, acute non-occlusive thrombus in the proximal and distal right internal iliac artery, and total occlusion of the right popliteal artery below the knee.

**Figure 2:**
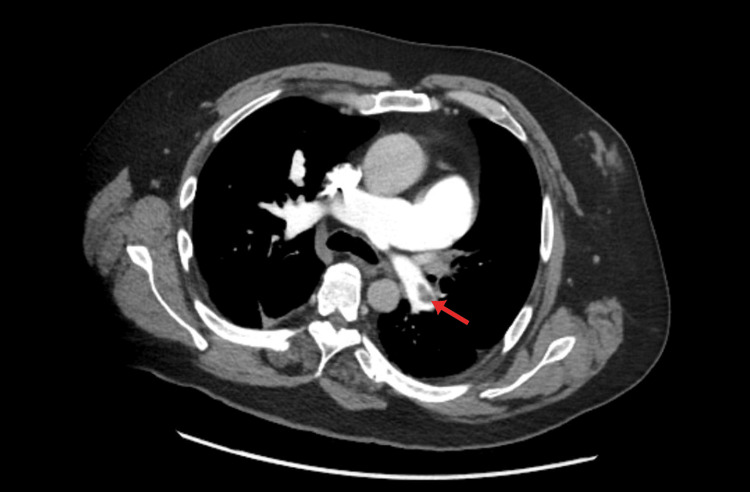
Axial computed tomography angiography showing a segmental left pulmonary embolus. Repeat computed tomography angiography of the chest, abdomen, pelvis, and run-off confirming the presence of a pulmonary embolus (red arrow), suggesting an underlying concurrent venous thromboembolism process.

**Figure 3:**
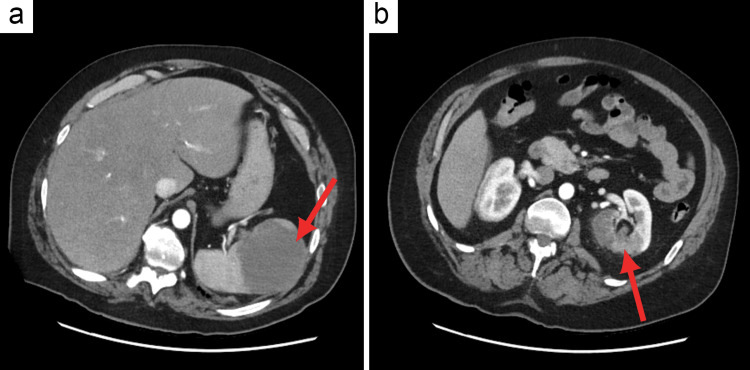
Computed tomography angiography of the patient’s abdomen. Repeat computed tomography angiography of the chest, abdomen, pelvis, and run-off confirming the presence of additional infarcted sites (red arrow), suggesting a multi-organ thromboembolic involvement. (a) Splenic infarction. (b) Left renal infarction.

Additionally, segmental and subsegmental pulmonary emboli were identified (Figure 2), involving the left upper lobe, lingula, and left lower lobe, as well as subsegmental pulmonary emboli in the right lower lobe. Other notable findings included an enlarged pulmonary outflow tract and pulmonary arteries suggestive of pulmonary hypertension, minimal aortic and great vessels plaque, right heart enlargement, and bowing of the septum with an elevated right ventricle to left ventricle (RV:LV) ratio of 1.3, suggestive of right heart strain, an indeterminate hypoattenuating lesion in the spleen possibly representing an infarct, and a left renal infarct (Figure 3).

These multiple CTA findings aligned with either a cardioembolic source or a paradoxical embolism, thus prompting further investigation into the source. An agitated saline study for intracardiac shunting was negative on transthoracic echocardiography (TTE), ruling out a paradoxical embolism as a potential cause of this patient’s presentation. The TTE also reported a biplane left ventricular ejection fraction (LVEF) of 68%, severely dilated right ventricle and right atrium, severely reduced right ventricular systolic function, tricuspid regurgitation, and moderately elevated pulmonary artery pressure (right ventricular systolic pressure moderately measuring 47 mmHg). These findings, along with the findings of CTA, further supported the presence of right ventricular dysfunction as a consequence of pulmonary hypertension, possibly due to the combined effects of the patient’s history of repaired congenital heart disease and the untreated OSA.

The presence of right ventricular dysfunction and high troponin levels, despite the patient’s low initial PESI score, qualifies the case as intermediate-low risk for PE severity and early (in-hospital or 30-day) mortality. For the early diagnosis of hemodynamic decompensation or collapse in such instances, close monitoring is advised [4].

The patient’s ALI was classified as Rutherford IIa, and he was admitted and started on intravenous fluids, pain management, and heparin. Because of the patient’s substantial comorbidities and inadequately optimized cardiovascular risk factors, urgent endovascular revascularization was recommended. Surgical revascularization was difficult due to the complicated anatomy of the lower extremity vasculature and the high embolic burden. The decision was also influenced by the facility’s expertise with endovascular procedures.

Attempts to gain access in the right brachial and right radial arteries were unsuccessful due to likely arterial occlusions with no pulsatile flow, suggesting chronic peripheral arterial disease (PAD) and collateralization. Nurses were unable to obtain blood pressure readings in the left upper extremity, and access attempts in the left brachial and radial arteries also failed, further supporting the presence of diffuse chronic PAD, which could be a factor in the acute presentation. Successful access was eventually obtained in the left common femoral artery, and a 6-French sheath was placed, later exchanged for a 7-French sheath. Peripheral angiography revealed occlusion of the right external and common iliac arteries.

**Figure 4:**
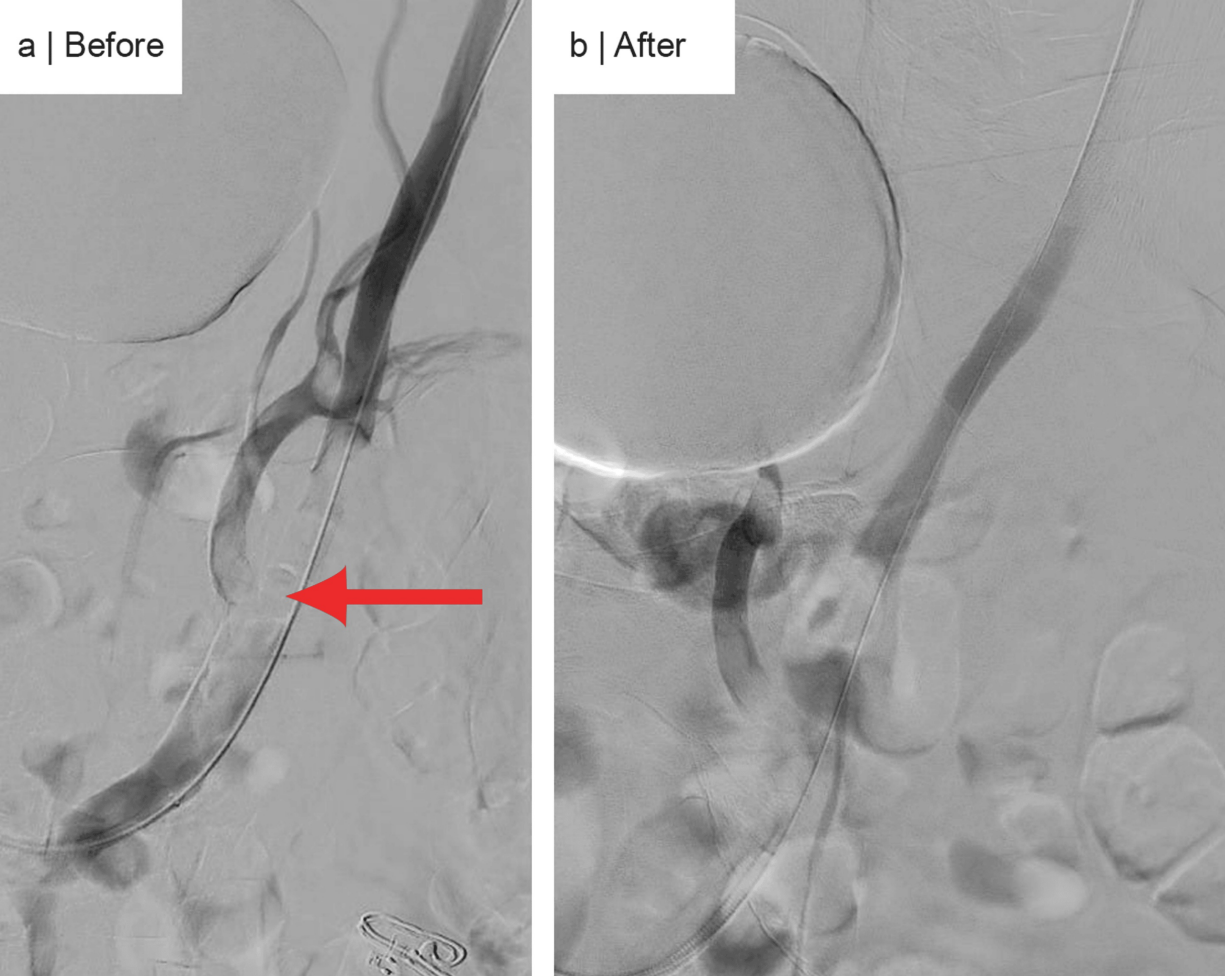
Peripheral angiography showing pre- and post-aspiration (Penumbra) thrombectomy and balloon angioplasty of the right common and external iliac arteries. (a) Pre-thrombectomy: a short-segment occlusion of the proximal right external and internal iliac arteries. (b) Post-thrombectomy: 0% residual stenosis in the proximal right external and internal iliac arteries.

Aspiration thrombectomy was performed using the (Penumbra) device with multiple runs, followed by balloon angioplasty with a 7.0 × 80 mm (Ultraverse) balloon, resulting in 0% residual stenosis (Figure 4). However, a heavy thrombus burden persisted in the right popliteal artery with no flow below the knee. Multiple additional runs of aspiration thrombectomy with (Penumbra) failed to restore adequate flow. A long 5-French × 110 cm sheath was placed through the 7-French sheath, and a (McNamara) catheter was successfully positioned to initiate a tissue plasminogen activator (tPA) infusion in the catheterization lab. The patient showed improved flow and foot movement post-procedure.

Catheter-directed thrombolysis (CDT) with overnight tPA infusion was chosen due to the patient’s classification as Rutherford IIa ALI. Patients in this category benefit most from CDT, as early thrombolysis can restore perfusion while minimizing surgical risks [12].

The patient was transferred to the critical care unit (CCU) for overnight tPA infusion at 2 mg/hour and heparin drip at 1,000 units/hour through the 7-French sheath. PTT, coagulation profile, fibrinogen, and fibrinogen degradation products were all serially measured every six hours, and the patient was closely monitored.

**Figure 5:**
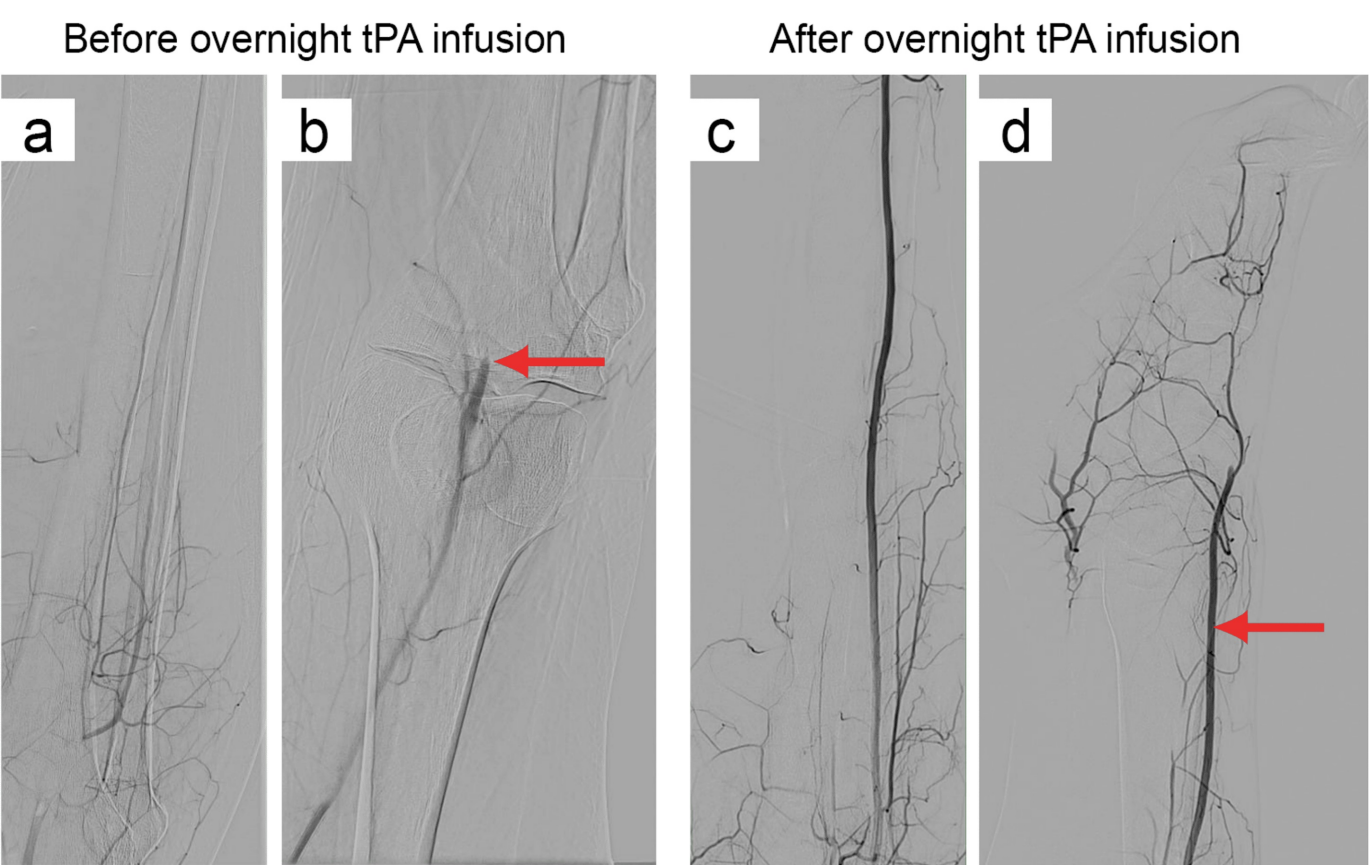
Peripheral angiography showing pre- and post-overnight tissue plasminogen activator (tPA) infusion of the right popliteal artery and its flow below the knee. Results of overnight tPA infusion, demonstrating pre- and post-treatment arterial flow. Pre-tPA infusion: (a) Heavy thrombus burden in the right popliteal artery. (b) Complete occlusion of the right popliteal artery below the knee (red arrow), with absent distal flow. Post-tPA infusion: (c) Restored flow in the right popliteal and anterior tibial arteries. (d) Reestablished perfusion in the right dorsalis pedis (red arrow).

The following day, the patient reported a drastic improvement in symptoms since the start of the tPA infusion. The right lower extremity was warm with improved pulses. The patient was brought back to the catheterization lab for a relook angiogram. Access in the left common femoral artery had already been obtained, and the short 7-French sheath and the long 5-French sheath were exchanged for a long 7 × 110 cm sheath. The angiogram demonstrated excellent flow in the right anterior tibial artery into the right dorsalis pedis (Figure 5). However, there was a significant thrombus burden in the peroneal and posterior tibial arteries.

**Figure 6:**
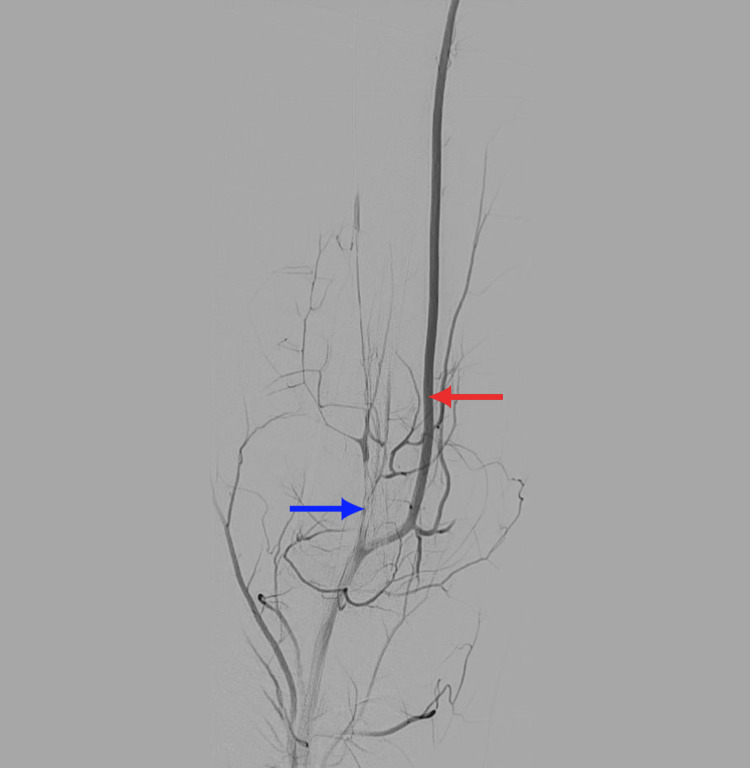
Peripheral angiography showing restored flow in the right anterior tibial artery and persistent thrombus burden in the peroneal and posterior tibial arteries after endovascular revascularization. Red arrow: Right anterior tibial artery. Blue arrow: Thrombus burden in the peroneal and posterior tibial arteries.

An attempt was made to wire the peroneal artery through the long 7-French sheath and perform balloon angioplasty, but the attempt was unsuccessful. A decision was made to obtain pedal access in the right posterior tibial artery under ultrasound guidance. A 4-French sheath was advanced over the wire, and balloon angioplasty was performed using 2.0 mm and 4.0 mm balloons, which resulted in improved flow but a persistent heavy thrombus burden (Figure 6). It was decided to stop further tPA infusion and forego further intervention due to the lack of neurovascular impairments, the positive clinical results, and the relook angiogram’s positive results, indicating remission of ALI symptoms.

The patient tolerated the procedure with no immediate complications. He was transitioned off heparin and started on rivaroxaban 15 mg twice daily for three weeks, followed by 20 mg daily for a total of six months. Rivaroxaban, a direct oral anticoagulant (DOAC), was chosen because of the patient’s potential non-compliance with warfarin therapy, which includes neglecting follow-up visits and checking INR levels, and due to the simpler therapeutic approach of DOACs.

The next day, the patient experienced complete resolution of his right lower extremity pain and was transferred back to the medical ward. Plans were made for repeat TTE to further evaluate possible cardioembolic sources and cardiac magnetic resonance imaging (CMR) to assess the degree of right ventricular dysfunction following the TOF repair.

Repeat TTE revealed no pulmonic valve regurgitation or stenosis post-repair, and no left ventricular thrombus with intravenous contrast. Furthermore, it showed multiple improvements in comparison with the previous TTE with moderately dilated right ventricle, moderately reduced right ventricular systolic function, and normal pulmonary artery pressure (right ventricular systolic pressure moderately measuring 24 mmHg). CMR demonstrated a dilated right atrium and right ventricle, with no intracardiac thrombus. An interventricular septal bounce during diastole was observed, further supporting the presence of elevated right heart pressures. As the results of TTE and CMR were believed to be adequate for evaluating intracardiac shunting and structural abnormalities, transesophageal echocardiography (TEE) was not performed.

During hospitalization, the patient required 4 L/minute of supplemental oxygen via a nasal cannula due to hypoxic respiratory failure, likely secondary to chronic pulmonary hypertension, which is most likely attributed to the untreated OSA and the history of repaired TOF in the absence of intracardiac shunting. Additionally, given the patient’s extensive smoking history, chronic obstructive pulmonary disease (COPD) could be considered, and tiotropium bromide (two puffs daily) was initiated.

Extensive hypercoagulability and occult malignancy screenings, including tests for factor V Leiden, lupus anticoagulant, beta-2-glycoprotein I, anticardiolipin antibodies, carcinoembryonic antigen, and prostate-specific antigen, were negative. Hemoglobin A1C was measured and found to be 5.3%.

The patient’s respiratory status improved five days later and was discharged with instructions for home supplemental oxygen therapy at 4 L/minute during ambulation. Additional discharge recommendations included the use of a rolling walker, an elective right heart catheterization to further assess right ventricular dysfunction, an elective overnight sleep study to evaluate OSA, and a scheduled follow-up for pulmonary hypertension with pulmonary function testing. To check for potential paroxysmal atrial fibrillation, Holter monitoring was also advised.

**Figure 7:**
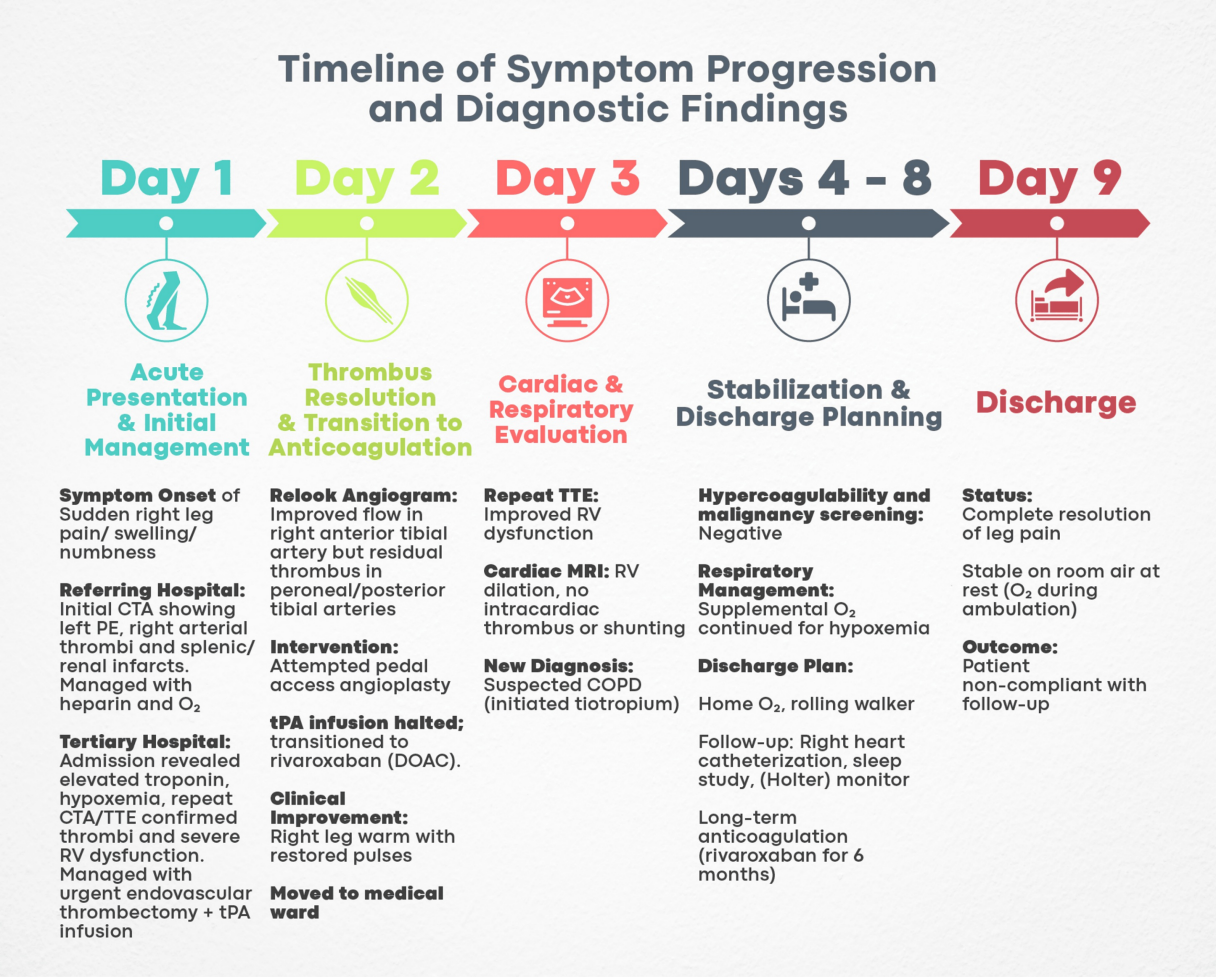
Timeline of symptom progression and diagnostic findings. CTA: computed tomography angiography; PE: pulmonary embolism; TTE: transthoracic echocardiogram; RV: right ventricle; tPA: tissue plasminogen activator; DOAC: direct oral anticoagulant; COPD: chronic obstructive pulmonary disease

Because of the patient’s unprovoked multisite embolism and the negative hypercoagulable workup, secondary prophylaxis after six months was taken into consideration. However, the patient did not follow up with the discharge instructions or start long-term preventive therapy because they did not show up for their planned follow-up visits. Figure 7 provides a simple illustration of the timeline of symptom progression and findings for our case.

## Discussion

This case highlights a complex scenario of concurrent systemic thromboembolic conditions, such as ALI and PE. This scenario requires prioritizing the most life-threatening condition while managing co-existing conditions, balancing anticoagulation-related bleeding risks, and selecting the most appropriate intervention based on the patient’s overall clinical picture.

Long-term vascular complications should also be an ongoing discussion when managing adult patients with repaired CHD. ALI is an alarming feature of this case and should always prompt urgent diagnosis and intervention. It is a critical, potentially life-threatening condition that presents in patients with multiple medical comorbidities and risk factors, such as diabetes mellitus, hypertension, hyperlipidemia, obesity, and smoking, most of which were at play in this case [8].

ALI threatens limb viability and places the patient at risk of many systemic complications, such as acid-base imbalances, electrolyte disturbances, and other metabolic abnormalities, which become more prominent in cases with delayed reperfusion via ischemia-reperfusion injury. Prolonged ischemia triggers the release of inflammatory mediators and intracellular contents such as lactic acid and electrolytes, which, upon reperfusion, overwhelm the body and cause metabolic acidosis and disruption of electrolyte equilibrium [8,13].

ALI may occur due to acute-on-chronic deterioration of limb perfusion in patients with pre-existing PAD, as suggested in this case by the procedural evidence of bilateral upper extremity arterial occlusions and collateralization. On the other hand, the abrupt onset of ALI, such as that manifested in this case, suggests an embolic source in a previously asymptomatic patient [13,14].

Symptoms of embolic ALI often progress to paralysis and irreversible ischemia within four to six hours due to absent collateral circulation. In contrast, thrombotic ALI in pre-existing PAD may evolve over days, supported by collaterals [8]. This patient’s sudden-onset symptoms (four-hour duration) aligned more with an embolic mechanism.

Common embolic sources include thrombus in the cardiac chambers, on valves, or from atherosclerosis of native arteries. This is often linked to atrial fibrillation, recent myocardial infarction, severe left ventricular dysfunction, endocarditis, or prosthetic valve leaflet thrombosis from inadequate anticoagulation. The rarer causes include venous obstruction (phlegmasia cerulea dolens), paradoxical emboli (across cardiac septal defects), vasculitides, such as Raynaud’s disease for the upper extremity and Buerger’s disease (thromboangiitis obliterans) for the lower extremities, and hypercoagulable states [8,14].

Another critical embolic event is PE, a potentially fatal manifestation of VTE that presents various diagnostic and therapeutic challenges. The co-occurrence of such venous pathology with an arterial event supports a shared pathophysiological mechanism, whereby unprovoked VTE can carry a fourfold increased risk of myocardial infarction within 10 years in a Canadian cohort study, for example [15].

Some factors are particularly relevant in CHD and may underlie this shared mechanism. These include systemic hypercoagulability (e.g., obesity, smoking, and hypertension), paradoxical embolism via residual intracardiac shunts, and hemodynamic stasis from right ventricular dysfunction or immobility. Additionally, endothelial dysfunction and chronic inflammation, both implicated in arterial and venous thrombosis, may serve as underlying contributors to this overlap. Together with genetic predispositions, these processes demonstrate the intricate relationship between venous and arterial diseases [7,10].

CTA findings of splenic and renal infarcts, along with ALI, suggest a cardioembolic source, as emboli from the heart or large vessels can enter the systemic circulation. On the other hand, the coexistence of ALI and PE in this patient supports a systemic thromboembolic predisposition rather than isolated arterial or venous pathology. The non-occlusive DVT, though less severe, further supports a systemic prothrombotic state that warrants investigation in repaired CHD patients.

In this case, an extensive workup was performed, including TTE with bubble contrast study, CMR, and a thrombophilia screening panel. All of these excluded a paradoxical embolic source and the presence of a prothrombotic state. This raises questions about the role of TOF-related hemodynamic abnormalities and their potential contribution to cardioembolism.

Chronic complications following TOF repair include pulmonary regurgitation, residual stenosis, and arrhythmias [10]. Another long-term outcome of surgically repaired CHD is persistent or recurrent pulmonary arterial hypertension, which is associated with poor prognosis [16]. In this case, multiple investigations suggested an element of pulmonary hypertension, potentially linked to the patient’s untreated OSA or an undiagnosed COPD. While ventricular arrhythmias are well-characterized in TOF survivors [5,11], atrial arrhythmias, such as atrial fibrillation, are increasingly recognized in aging CHD populations and may be underrecognized in this cohort [5,6,10,11]. These arrhythmias likely arise from structural substrates, including right atrial dilation secondary to chronic volume overload (e.g., pulmonary regurgitation) or scarring from prior surgical repairs (e.g., atriotomies). Such anatomical and electrical remodeling creates arrhythmogenic substrates that predispose to atrial tachyarrhythmias, even in the absence of documented episodes during routine monitoring. Notably, atrial arrhythmias are a leading cause of hospitalization in adults with CHD and may contribute to thromboembolic risk via blood stasis. This underscores the importance of extended rhythm surveillance (e.g., ambulatory Holter monitoring) in TOF survivors, particularly as they age.

To date, there is limited research on the risk of thromboembolism in repaired CHD patients in the absence of paradoxical embolism. Prospective cohort studies focusing on aging repaired CHD patients and inclusion in thromboembolic registries are needed to clarify risks and guide anticoagulation strategies. While one meta-analysis found an increased risk of cardiovascular disease in adults with CHD, it remains unclear whether this reflects confounding risk factors or an intrinsic relationship between CHD and thromboembolism [17].

A sizeable Swedish case-control study found that, over a mean follow-up of approximately 16 years from birth, patients with CHD had a threefold increased risk of VTE compared to controls, with those having conotruncal defects (such as TOF) at the highest risk [18]. The study also reported that VTE incidence rose with age, with the cumulative incidence increasing, most notably around 40 years of age for both CHD patients and controls. However, the study had a relatively young sample (mean age ~17.4 years) and did not differentiate between operated and unoperated patients. Many unoperated patients are not followed into adulthood due to a lower likelihood of late complications, given their relatively simple and minor lesions [19]. Consequently, an undivided analysis that includes younger patients may obscure a focused evaluation of VTE risk in adult CHD surgery survivors.

ALI management involves either endovascular or surgical interventions, with the latter reserved for severe ischemic limbs. The choice between the two modalities is also influenced by vascular anatomy, the patient’s risk factors, and the available local expertise. For this case with Rutherford IIa ALI, endovascular intervention with catheter-directed thrombolysis is often preferred due to its minimally invasive nature and lower morbidity. Immediate anticoagulation with heparin was also initiated to prevent thrombus propagation, per standard practice [12,13]. Surgical revascularization was unfavorable given the patient’s complex vascular anatomy and the multiple coexisting morbidities. The choice was also driven by the presence of distinguished clinical expertise in endovascular thrombectomy.

Anticoagulation therapy for both ALI and PE is essential but must be carefully managed to avoid bleeding complications, especially with interventional treatments. Heparin is initially used due to its short half-life, allowing quick adjustments. Once stabilized, the patient was switched to a DOAC rather than warfarin due to compliance issues and the need for consistent follow-up.

Coordinated input from interventional cardiology (endovascular revascularization), hematology (anticoagulation strategy), and pulmonology (PE/respiratory management) was critical to balancing competing priorities in this case by addressing limb-threatening ALI while mitigating bleeding risks from thrombolytics and anticoagulants. This multidisciplinary framework highlights actionable lessons for clinical practice: early involvement of specialists optimizes triage of life-threatening events, and repaired TOF patients require vigilant surveillance for thromboembolic risks.

To provide a clear, stepwise overview of optimal treatment strategies for systemic thromboembolic disease, we have developed Table 1, which summarizes the key recommendations from the American Society of Hematology (ASH) 2020 Guidelines for the Treatment of Deep Vein Thrombosis and Pulmonary Embolism [20]. This table delineates the management phases, from initial risk assessment and treatment initiation through primary treatment to secondary prevention, and emphasizes the importance of a multidisciplinary approach to optimize patient outcomes.

**Table 1:** Management of systemic thromboembolic disease based on ASH 2020 guidelines. ASH: American Society of Hematology; PESI: Pulmonary Embolism Severity Index; PE: pulmonary embolism; LMWH: low-molecular-weight heparin; UFH: unfractionated heparin; DOAC: direct oral anticoagulant; VKA: vitamin K antagonists; INR: international normalized ratio

Phase	Time frame	Key interventions and considerations
Initial management	First 5–21 days	Risk stratification using clinical scores (e.g., PESI for PE)
		Determination of treatment setting (home vs. hospital)
		Initiation of anticoagulation (LMWH/UFH)
		Consideration of thrombolytic therapy for patients with hemodynamic compromise
Primary treatment	3–6 months	Continued therapeutic anticoagulation
		Transition to oral anticoagulants (DOACs or VKA)
		Monitoring for efficacy and safety (e.g., INR monitoring for VKA therapy)
Secondary prevention	Indefinite (if indicated)	Evaluation of the risk–benefit ratio for long-term anticoagulation
		Decision based on patient-specific factors (e.g., recurrence risk, bleeding risk)
		Regular reassessment and potential adjustment of therapy

## Conclusions

This case highlights the rare co-occurrence of ALI and PE in a patient with repaired TOF. Key issues include distinguishing embolic from thrombotic ALI, balancing anticoagulation risks, and addressing congenital factors such as right ventricular dysfunction and arrhythmogenic changes from prior surgeries. Our case, which demonstrated clear arterial occlusions, subclinical chronic vascular disease, and an absence of overt embolic sources, directly informs these challenges. Future research should prioritize multicenter, prospective studies to define thromboembolic risks in aging congenital heart disease cohorts. Additionally, studies should evaluate the effectiveness of empiric anticoagulation in high-risk subgroups (e.g., right atrial dilation, subclinical arrhythmias) and assess the need for standardized multidisciplinary care protocols. These should involve adult congenital cardiology for long-term hemodynamic surveillance, hematology for personalized anticoagulation management, vascular surgery for limb revascularization, and advanced imaging specialists (CMR/TEE) for detecting occult thrombi or shunts.
